# Analysis of Pulse Arrival Time as an Indicator of Blood Pressure in a Large Surgical Biosignal Database: Recommendations for Developing Ubiquitous Blood Pressure Monitoring Methods

**DOI:** 10.3390/jcm8111773

**Published:** 2019-10-24

**Authors:** Joonnyong Lee, Seungman Yang, Saram Lee, Hee Chan Kim

**Affiliations:** 1 Seoul National University Hospital Biomedical Research Institute, Seoul 03080, Korea; joonnyonglee@melab.snu.ac.kr (J.L.); hommelee@snu.ac.kr (S.L.); 2 Interdisciplinary Program in Bioengineering, Seoul National University Graduate School, Seoul 03080, Korea; ysmgreen@melab.snu.ac.kr; 3 Department of Biomedical Engineering, College of Medicine, Seoul National University, Seoul 03080, Korea; 4 Institute of Medical & Biological Engineering, Medical Research Center, Seoul National University, Seoul 03080, Korea

**Keywords:** blood pressure monitoring, pulse arrival time, pulse transit time, pulse wave velocity, biosignal database, ubiquitous healthcare, hypertension, cardiovascular monitoring

## Abstract

As non-invasive continuous blood pressure monitoring (NCBPM) has gained wide attraction in the recent decades, many pulse arrival time (PAT) or pulse transit time (PTT) based blood pressure (BP) estimation studies have been conducted. However, most of the studies have used small homogeneous subject pools to generate models of BP based on particular interventions for induced hemodynamic change. In this study, a large open biosignal database from a diverse group of 2309 surgical patients was analyzed to assess the efficacy of PAT, PTT, and confounding factors on the estimation of BP. After pre-processing the dataset, a total of 6,777,308 data pairs of BP and temporal features between electrocardiogram (ECG) and photoplethysmogram (PPG) were extracted and analyzed. Correlation analysis revealed that PAT or PTT extracted from the intersecting-tangent (IT) point of PPG showed the highest mean correlation to BP. The mean correlation between PAT and systolic blood pressure (SBP) was −0.37 and the mean correlation between PAT and diastolic blood pressure (DBP) was −0.30, outperforming the correlation between BP and PTT at −0.12 for SBP and −0.11 for DBP. A linear model of BP with a simple calibration method using PAT as a predictor was developed which satisfied international standards for automatic oscillometric BP monitors in the case of DBP, however, SBP could not be predicted to a satisfactory level due to higher errors. Furthermore, multivariate regression analyses showed that many confounding factors considered in previous studies had inconsistent effects on the degree of correlation between PAT and BP.

## 1. Introduction

According to the World Health Organization (WHO) cardiovascular diseases (CVD) are the leading causes of mortality around the globe, representing a heavy socioeconomic burden for the affected individuals [1]. Hypertension, or abnormally high blood pressure (BP), is the most important risk factor for CVD and is often a therapeutic target for CVD patients [2,3,4,5,6,7]. Although hypertension can be prevented through careful management of BP [8], there has been a lack of adequate devices for the early diagnosis and prevention of hypertension.

Recent studies have shown that a single discrete BP measurement at the clinic can be misleading [8,9,10], and that methods which can observe changing patterns of BP, such as ambulatory BP monitoring (ABPM), are required for the accurate diagnosis of hypertension [10,11,12]. However, the periodic oscillometric operation of ABPM devices leads to a lowered quality of life for the users [13,14], and due to this inconvenience, it is not optimal for non-invasive continuous BP monitoring (NCBPM).

In light of this need, multiple indirect approaches to BP estimation have been developed throughout the last few decades, and pulse wave velocity (PWV) based methods have gained the most interest [15,16,17,18,19,20,21,22,23,24]. Starting with Geddes et al. in 1981 [17], a wide range of studies using PWV and related parameters have been published. PWV is defined as the velocity at which an arterial pulse travels in an artery from a proximal point to a distal point and is often approximated using its surrogate pulse transit time (PTT), which is the time delay for the pulse to travel between two different arterial sites. Through the well-known arterial compliance modelling of BP [25,26], PTT is inversely related to BP with a negative correlation to BP. The most commonly used method of BP estimation using this concept involves the extraction of pulse arrival time (PAT) defined as the time delay from the R-peak of the electrocardiogram (ECG) to a peak of the finger photoplethysmogram (PPG) [27]. However, many alternative modalities of PAT or PTT measurement have been proposed, and the results vary as widely.

Though some studies promise PAT and/or PTT as the base for the future of BP measurement [16,18,24,28,29,30], other studies show questionable results [31,32]. Furthermore, results from long-term studies show decreasing accuracy [33], and the small sample size of the studies render the results inconclusive. While a few studies have used diverse groups of subjects [34], most of these studies only provide results based on small homogeneous subject pools with varying biosignal measurement modalities, and these issues may account for the discrepancies between the study results. Additionally, in order to obtain a wider range of BP, these studies often use certain types of interventions to induce hemodynamic changes, but these interventions are not representative of the full range of physiological causes that can result in a change in BP. Although it is difficult to argue against using non-invasive interventions to obtain demonstrable results at an experiment level, this also raises questions regarding the real world applicability of methods developed from such experiments.

Due to these limitations, researchers have turned to large biosignal databases for the development and analysis of BP estimation models [35,36,37,38,39], with the most focus on the Physionet Medical Information Mart for Intensive Care (MIMIC) database due to its large size and accessibility [40,41]. However, these databases are often collected from multiple sources, which restricts analyzing the dataset as a whole, since the variability in biosignal measurement modalities may cause time-domain inconsistencies in parameter extraction. In a recent study, Liang et al. demonstrated that inter-waveform analyses using MIMIC led to erroneous conclusions [42]. Therefore, to this day, it is unclear whether BP estimation methods based on PAT or PTT can be applied to large subject pool with varying subject characteristics.

In this paper, a large open biosignal database of surgery patients is analyzed to address the fundamental question regarding the applicability of PAT or PTT to BP estimation. The data used in this study was not collected for any specific purpose, such as PAT based BP modelling, and is therefore representative of real world situations in which PAT and PTT respond dynamically to BP depending on the underlying hemodynamic mechanism. First, the database and the signal measurement modalities are introduced and the method of biosignal feature extraction is described. Then, the analysis method is presented in detail. Lastly, the results of the analysis are presented and discussed.

## 2. Methods

### 2.1. VitalDB Database

**Figure 1:**
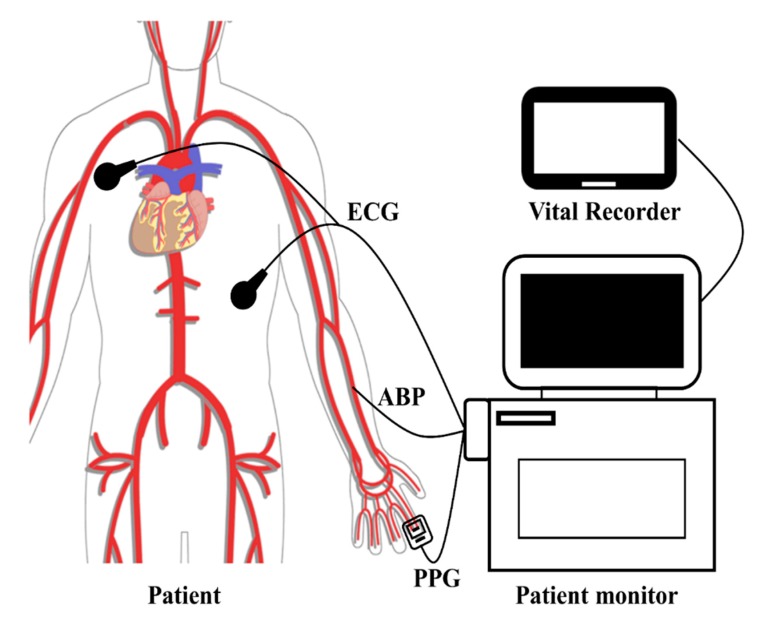
Experimental setup of VitalDB data collection protocol. ECG, electrocardiogram; PPG, photoplethysmogram; ABP, arterial blood pressure.

The data used in this study is a part of the VitalDB data bank, which is an open access public dataset of intraoperative vital signs and biosignals collected by the Seoul National University Hospital Department of Anesthesia using the Vital Recorder program [43]. The Vital Recorder program is a free research tool for recording of time-synchronized physiological data from multiple intraoperative devices and patient monitors. The experimental setup for collecting waveform data of VitalDB is shown in Figure 1. The collected VitalDB data includes raw waveforms of ABP, ECG, and PPG obtained from a commercial patient monitor device (SOLAR 8000M, GE, Milwaukee, WI, USA) down-sampled to 100Hz. The ECG waveform is measured using a standard lead II setup, ABP waveform is measured from the radial artery, and PPG waveform is measured from the finger. All recordings are associated with a subject ID, and each ID is matched with demographic data (e.g., age, gender, BMI, etc.), surgery and anesthesia data (e.g., surgery type, operation, type of anesthetic used, etc.), and preoperative data (e.g., the presence of hypertension, blood test hemoglobin measurement, etc.).

### 2.2. Data Selection

**Figure 2:**
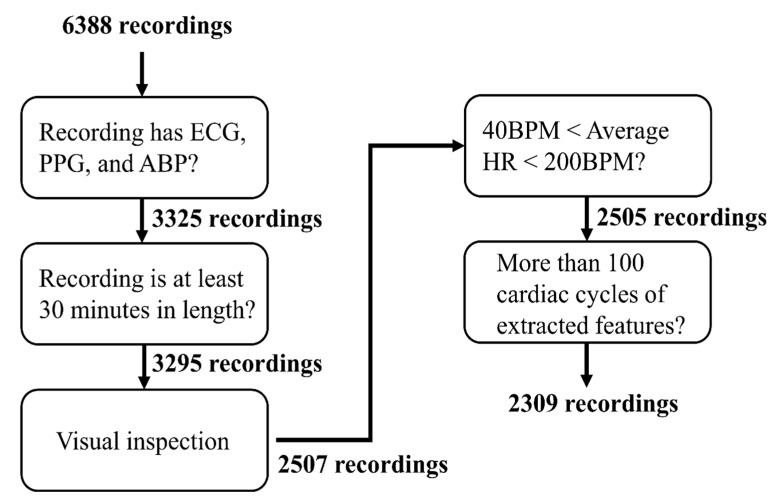
Outline of the data exclusion process for the selection of usable data from the VitalDB data bank and data pre-processing. ECG, electrocardiogram; PPG, photoplethysmogram; ABP, arterial blood pressure; HR, heart rate; BPM, beats per minute; SBP, systolic blood pressure.

The VitalDB data bank contains biosignal recordings from a total of 6388 patients undergoing various types of surgeries at the Seoul National University Hospital. Data loading, data selection, and feature extraction were performed automatically using MATLAB (MATLAB 2018b; Mathworks, Natick, MA, USA). The data used in this study were selected from the database following a certain set of exclusion criteria shown in Figure 2. First, the recordings were checked for ECG, PPG, and ABP. Second, recordings under 30 minutes were removed. Third, recordings were visually checked for saturation and other distortions to remove corrupted portions of the recording or to remove the recording as a whole. Fourth, recordings with an average heart rate below 40 BPM or above 200 BPM, as found using the Pan-Tompkins algorithm [44], were removed. Lastly, recordings with a total number of analyzable data under 100 cardiac cycles were removed following feature extraction. In total, 2309 recordings were selected from a pool of 6388 recordings.

### 2.3. Pre-Processing and Feature Extraction

**Figure 3:**
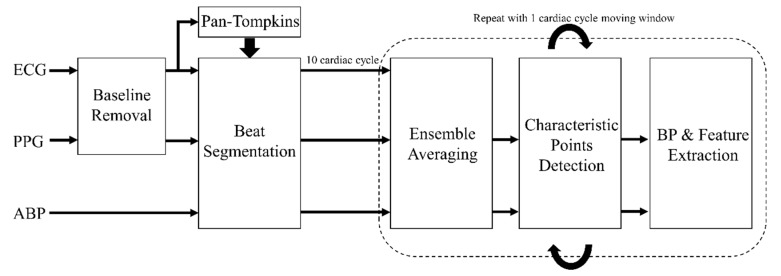
Process of BP and feature extraction from ECG, PPG, and ABP waveforms. ECG, electrocardiogram; PPG, photoplethysmogram; ABP, arterial blood pressure.

In order to verify that features such as PAT and PTT are valid markers of blood pressure, features and reference blood pressure values from ABP, ECG, and PPG waveforms were extracted for each recording. The detailed BP and feature extraction process is shown in Figure 3. First, the low frequency artefacts of ECG and PPG waveforms were removed using non-linear filtering [45]. Then, the Pan-Tompkins algorithm was used to detect ECG R-peaks, which were used to separate the waveform data into cardiac cycles for ensemble-averaging. In order to improve the signal-to-noise ratio (SNR) and to accentuate the waveform features, ABP and PPG waveforms between 10 consecutive R-peaks were ensemble averaged to obtain the average waveforms of ABP and PPG. This process was repeated for the whole recording with a moving window width of one cardiac cycle.

**Figure 4:**
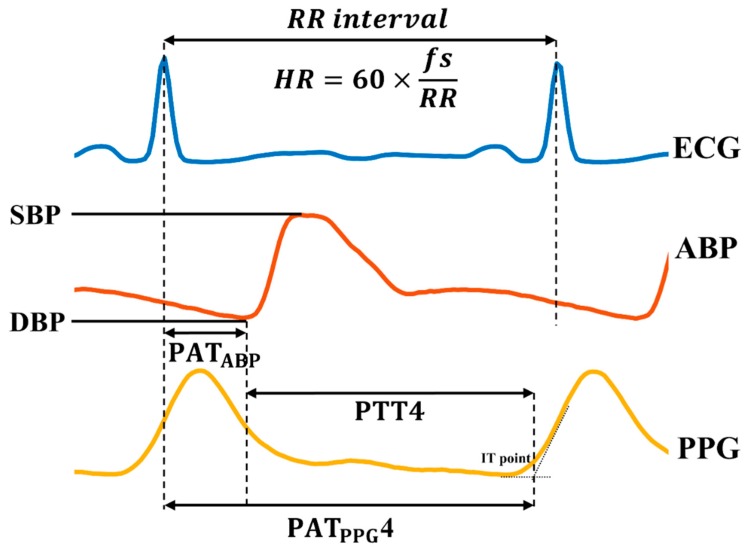
BP and features labeled on ECG, PPG, and ABP waveforms. Among the four characteristic points of PPG, only the IT point is labeled. SBP, systolic blood pressure; DBP, diastolic blood pressure; ABP, arterial blood pressure; ECG, electrocardiogram; PPG, photoplethysmogram; PAT, pulse arrival time; PTT, pulse transit time; IT, intersecting tangent; HR, heart rate; fs, sampling frequency; RR, the interval between successive ECG R-peaks.

After ensemble averaging, the peaks and the valleys of the ABP were detected. For the PPG, the valleys, peaks, maximum derivatives (or the point of maximum slope), and intersecting-tangent (IT) points [46] (or the intersection between the tangent lines of the maximum derivative and the diastolic minimum) were detected (Figure 4). If the values for the all detected points in a given cardiac cycle were within pre-set time ranges (which were determined through analysis of the data set as a whole and the physiological ranges for each point), features and reference BP values were reserved for further analyses.

SBP and DBP were obtained as the values at the peak and the valley points of the ABP, mean blood pressure (MBP) was calculated as the average value of one cardiac cycle of ABP waveform, and pulse pressure (PP) was calculated as the difference between SBP and DBP. Time-based BP estimation parameters were extracted from each cardiac cycle using the R-peak point of ECG, valley point of ABP, and four feature points of PPG. PAT_ABP_ was derived from the time difference between the ECG R-peak and the valley point of ABP, PAT_PPG_ was derived from the time difference between the ECG R-peak and one of the characteristic points of PPG, and PTT was derived from the time difference between the valley point of ABP and one of the characteristic points of PPG. As four kinds of characteristic points from the PPG waveform were used, four different PAT_PPG_ (PAT_PPG_1, PAT_PPG_2, PAT_PPG_3, and PAT_PPG_4) and PTT (PTT1, PTT2, PTT3, and PTT4) values were extracted for each cardiac cycle (‘1’ corresponds to the valley, ‘2’ corresponds to the peak, ‘3’ corresponds to the maximum derivative, and ‘4’ corresponds to the IT points of PPG). The mean and the standard deviations of all PAT and PTT values are tabulated in Supplementary Table S1.

Due to the saturations and noises present in the ABP and PPG waveforms, the features and the reference BP values were tested against the following conditions:

- Is the extracted SBP greater than 50 mmHg and less than 250 mmHg?
- Is the extracted DBP greater than 30 mmHg and less than 160 mmHg?
- Is the extracted PP greater than 10 mmHg?
- Is the change in the extracted BP (SBP or DBP) during the previous 5 s interval less than 30 mmHg?
- Is the extracted PAT_ABP_ greater than 70 ms and less than 250 ms?
- Is the change in the extracted features (PAT or PTT) during the previous 5 s interval less than 300 ms?

If the above conditions were not met, the feature and BP values were excluded from further analyses. The range of PAT_ABP_ was determined by considering the normal range of the pre-ejection period (PEP) and the normal range of PTT through the central arteries based on the results of previous studies [27,47]. All features and BP values were then smoothed using a 20 s smoothing window. If the number of remaining features and BP values for a given subject was less than 100, the recording was excluded as shown in Figure 2. On average, 2935 cardiac cycles of data per subject were extracted. The demographic and BP characteristics of the 2309 subjects are shown in Table 1.

### 2.4. Feature Analysis

In order to evaluate the capacity of the extracted time-based features to estimate BP, correlation analysis was performed. The Pearson correlation coefficient was calculated between each feature and BP as followed:

(1) $$\rho \left(X,Y\right)=\frac{E\left[\left(X-\mu_{X}\right)\left(Y-\mu_{Y}\right)\right]}{\sigma_{X}\sigma_{Y}}$$

where *σ_X_* and *σ_Y_* are the standard deviations of *X* and *Y*, *μ_X_* and *μ_Y_* are the means of *X* and *Y*, and E is the expected value. The correlation coefficients between four reference BP values (i.e., SBP, DBP, MBP, and PP) and nine time-domain features (i.e., PAT_ABP_, four PAT_PPG_s, and four PTTs) for each subject data were obtained. The mean and the standard deviation values for the correlation coefficients were analyzed to determine the feature most associated with BP.

After correlation analysis, PAT_PPG_4 was used as a prediction parameter for BP (DBP, SBP) linear model using a total of 6,777,308 data pairs from 2309 subjects as followed:

(2) $$\mathrm{BP}=\alpha_{1}\cdot {\mathrm{PAT}}_{\mathrm{PPG}}4+\alpha_{0}$$

In order to calibrate the equation to each subject, the mean value of BP was subtracted from the BP values and the mean value of PAT was subtracted from the PAT values in a given recording prior to linear modelling. Then, the mean BP was added back to the zero-mean BP estimate output for each subject as *α*_0_ in the above equation.

To evaluate the performance of the BP linear model, the Pearson correlation coefficients between reference BP and BP estimates from each recording were calculated. Furthermore, to evaluate the models against the Association for the Advancement of Medical Instrumentation (AAMI) standards [48], the difference between the reference BP value and the estimated BP value was used to calculate mean error (ME) and standard deviation of the error (SDE) for each recording. To evaluate the efficacy of the model against the British Hypertension Society (BHS) BP monitor standards [49], the cumulative error percentages within 5, 10, and 15 mmHg were calculated. Lastly, the mean absolute difference (MAD) between the estimated BP and the reference BP was calculated for all subjects to validate the method against the IEEE Standard for Wearable Cuff-less BP Monitoring Devices (IEEE Standard 1708) [50]. The definitions of ME, SDE, and MAD are as followed:

(3) $$\mathrm{ME}=\frac{\sum_{i=1}^{n}\left(p_{i}-y_{i}\right)}{n}$$

(4) $$\mathrm{SDE}=\sqrt{\frac{\sum_{i=1}^{n}{\left[\left(p_{i}-y_{i}\right)-\mathrm{ME}\right]}^{2}}{n-1}}$$

(5) $$\mathrm{MAD}=\frac{\sum_{i=1}^{n}\left|p_{i}-y_{i}\right|}{n}$$

where *p_i_* is the estimated BP value, *y_i_* denotes the reference BP value, and *n* is the data size.

## 3. Results

**Figure 5:**
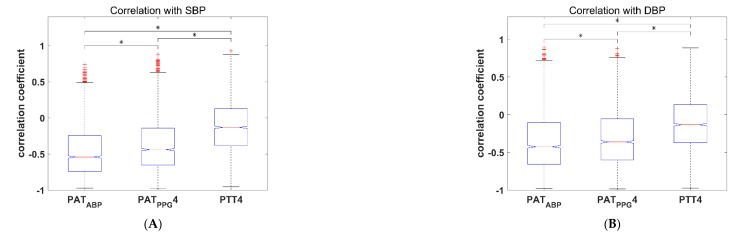

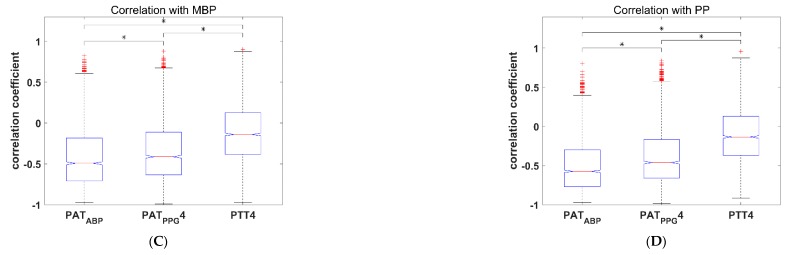
Box plots of the Pearson correlation coefficients between BP values and features of 2309 subjects. On each box, the central mark indicates the median, and the bottom and top edges of the box indicate the 25th and 75th percentiles, respectively. The whiskers extend to the most extreme data points not considered outliers, and the outliers are labeled with the ‘+’ symbol. (**A**) Box plot for SBP; (**B**) Box plot for DBP; (**C**) Box plot for MBP; (**D**) Box plot for PP. * *P* < 0.001 using the paired *t*-test. SBP, systolic blood pressure; DBP, diastolic blood pressure; MBP, mean blood pressure; PP, pulse pressure; PAT, pulse arrival time; PTT, pulse transit time.

The mean and standard deviation of the Pearson correlation coefficients between the four BP values (i.e., SBP, DBP, MBP, and PP) and the nine time-based features (i.e., PAT_ABP_, four PAT_PPG_s, and four PTTs) are shown in Table 2. Among the features, PAT_ABP_ showed the highest mean correlation to BP with -0.46 for SBP, −0.35 for DBP, −0.42 for MBP, and −0.50 for PP. Among the four PATs derived between ECG and PPG, PAT_PPG_4 showed the highest mean correlation to BP with −0.37 for SBP, −0.30 for DBP, −0.34 for MBP, and −0.39 for PP. In the case of PTT, PTT4 showed the highest mean correlation to BP, but showed very weak mean correlation with −0.12 for SBP, −0.11 for DBP, −0.12 for MBP, and −0.11 for PP. The box plots of the correlation coefficients between four BP values and three features (PAT_ABP_, PAT_PPG_4, and PTT4) are shown in Figure 5.

**Figure 6:**
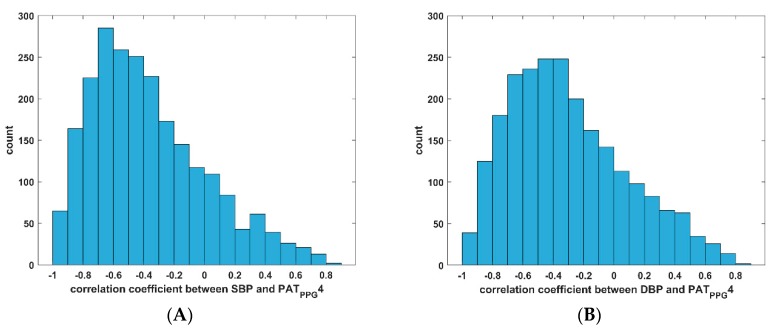
Histogram of the Pearson correlation coefficients between BP and PAT_ppg_4 of 2309 subjects. (**A**) Histogram of Pearson correlation coefficient between PAT_PPG_4 and SBP; (**B**) Histogram of Pearson correlation coefficient between PAT_PPG_4 and DBP. SBP, systolic blood pressure; DBP, diastolic blood pressure; PAT, pulse arrival time.

The distribution of the correlation coefficients between DBP, SBP, and PAT_PPG_4 are shown in Figure 6. For SBP, highest correlation values observed from the top 33% of the subjects are between −1 and −0.59, moderate correlation values observed from the middle 33% of subjects are between −0.59 and −0.27, and the lowest correlation values observed from the last 33% of subjects are between −0.27 and +1. Similar trends are seen with the correlation coefficient between DBP and PAT_PPG_4, with the 33% percentile value at −0.19 and the 66% percentile value at −0.52. Here, high correlation values are considered negative in reference to the theoretical inverse relationship between PAT and BP.

**Figure 7:**
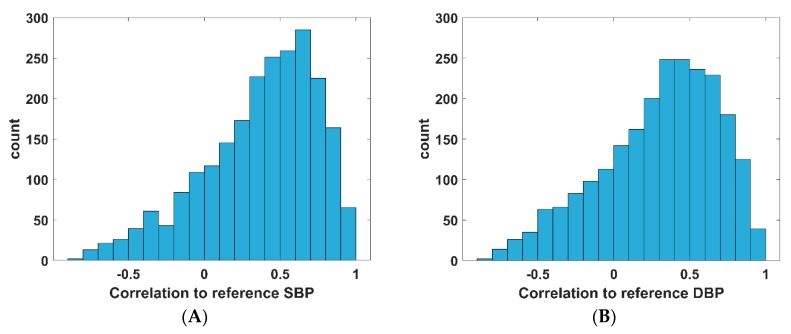
Correlation histograms for SBP and DBP estimation using the population BP linear model with 2309 recordings. (**A**) Correlation histogram for SBP estimation; (**B**) Correlation histogram for DBP estimation. SBP, systolic blood pressure; DBP, diastolic blood pressure.

The performance of the BP linear model is shown on Table 3 and Figure 7 (Bland Altman plots are seen on Supplementary Figure S1). The average correlation value between the reference SBP and the estimated SBP is 0.37, while it is 0.30 for DBP. The DBP estimation model satisfies the AAMI standards and is rated grade A against the BHS and IEEE standards. However, the SBP estimation model does not satisfy the AAMI standards and is rated grade C and grade D against the BHS and IEEE standards, respectively.

## 4. Discussion

In general, previous studies of PAT or PTT based BP estimation have used small homogeneous subject pools to generate models of BP based on particular interventions for induced hemodynamic change. However, these BP estimation models are not extendable for general use due to the nature of these experiments, and a comprehensive understanding of the general relationship between these predictors and BP was unavailable. In this study, biosignal data from a diverse group of 2309 surgical patients totaling over 6,500,000 cardiac cycles were analyzed. This is the first study to use such a large heterogeneous dataset to validate the potential of PAT and PTT as predictors of BP. The results of the analysis indicate that 1) in this experimental setup, the IT point of the PPG is the best to extract PAT or PTT for BP estimation and 2) PAT_ABP_ and PAT_PPG_ outperform PTT as an indicator for BP. Furthermore, due to the moderate degree of association between PAT_PPG_ and BP, a linear model of PAT_PPG_ based BP was able to output seemingly decent DBP estimate with a simple calibration method, but a more advanced method is necessary to accurately estimate SBP.

### 4.1. Pulse Arrival Time Versus Pulse Transit Time: Implications for Practical NCBPM

Contrary to prior studies [51,52,53], PAT was found to be a superior predictor of BP as compared to PTT. Many previous studies have shown that PTT, not PAT, is related to BP through arterial compliance, and have presented experimental evidence that support this idea. For example, in 2006, Payne et al. showed that due to the effect of PEP, changes in PAT may not be reflected with changes in BP, while PTT-based BP prediction is unaffected [52]. However, with regards to the development of a practical mobile NCBPM, PTT is often measured as the time difference between two PPG points (due to the small size and the low cost of PPG sensors), and this results in PTT that is derived from pulse propagation paths composed of both large arteries and peripheral arteries.

The smooth muscle interactions in peripheral arteries invalidate the assumptions of the arterial compliance model of BP [27] and, in contrast, disregarding the effect of PEP, PAT derived between ECG and ABP is derived from paths composing of large arteries that satisfy the above assumptions. As seen in Figure 1, the pulse propagation path for the measured PTT is between the radial artery and the peripheral arteries of the index finger, while the pulse propagation path for the measured PAT_PPG_ includes the path from the heart to the radial artery as well. Overall, the relative distance of the propagation path unaffected by smooth muscle interactions may be much greater for PAT_PPG_ as compared to PTT, and this is reflected in the correlation results shown in Table 2. The correlation values between BP and PAT_PPG_ are similar to the correlation values between BP and PAT_ABP_, while the correlation values between BP and PTT are significantly lower. This discrepancy indicates that pulse propagation timing mainly through large elastic arteries (i.e., PAT_PPG_ or PAT_ABP_) can be used for BP estimation, but increasing the ratio of peripheral arteries in the pulse propagation path (i.e., PPG based PTT) leads to the breakdown of the arterial compliance BP model.

Although previous studies have theorized that smooth muscle interactions in peripheral arteries would negatively affect PAT or PTT as predictors of BP, quantitative or qualitative analysis of this concept in regard to NCBPM was unavailable. These results provide evidence for the negative effect of smooth muscle interactions on the arterial compliance model-based estimation of BP, and provide insight into the potential modalities capable of measuring parameters related to BP. The measurement of PTT or PAT in large arteries using invasive means is in agreement with theory and may be ideal for BP estimation (as seen in the difference between correlation coefficients between BP and PAT_ABP_ or PAT_PPG_), but PTT derived from peripheral arteries using non-invasive means such as two PPGs are not adequate surrogates for BP estimation. To avoid the aforementioned issue, if two peripheral points of the body are to be used for PTT measurement for NCBPM, alternatives to PPG, such as mechanical pulse detection, should be used. Additionally, since the correlation values for PAT_ABP_ and PAT_PPG_ are similar, PAT derived from ECG R-peak and PPG may still have value in BP estimation as long as the distal point of pulse detection has a long enough pulse propagation path through large arteries. With consideration of the potential effect of PEP as described in [52,53], the ideal solution for NCBPM would involve a system which measures PTT from the aortic opening to a distal point using non-photoplethysmographic means, such as a system composed of seismocardiogram (SCG) and mechanical pulse detection at the wrist.

### 4.2. Variability of PAT-BP Relationship

In this dataset consisting of more than 2300 subjects, there is a moderate degree of correlation between BP and temporal inter-waveform predictors, but the correlation values varied widely with large standard deviation as shown on Table 2. Many previous studies have shown that age, gender, BMI, hypertension, and diabetes are associated with changes in PWV [54,55,56,57], which also may have contributed to the subject-specific variability of the relationship between BP and PAT. Since the data used in this study has the advantage that each recording is associated with the subject’s demographic data and preoperative data, it was possible to analyze the effect of various factors on the relationship between BP and PAT.

From the available data, age, gender, body max index (BMI), hypertension, and diabetes were analyzed as confounding factors on PAT-based BP estimation (effects on the slope and the correlation between PAT and BP, not on PAT or BP directly). First, the potential effect of the confounders on the slope between PAT and SBP were assessed by multivariate regression analysis. Age was the sole factor that had a significant effect on the slope between PAT and SBP, but the effect is miniscule as can be seen by the low β value. However, none of the risk factors had a significant effect on the correlation coefficients between SBP and PAT_PPG_4 as shown in Table 4. Similar results were found on the same multivariate regression analyses with DBP and PAT_PPG_4. Risk factors such as age, gender, BMI, hypertension, and diabetes could not determine the correlation between BP and PAT with any significant predictive power, and only age had a miniscule effect on the slope between PAT and BP. Therefore, the integration of these factors into a BP estimation model may not be needed.

**Figure 8:**
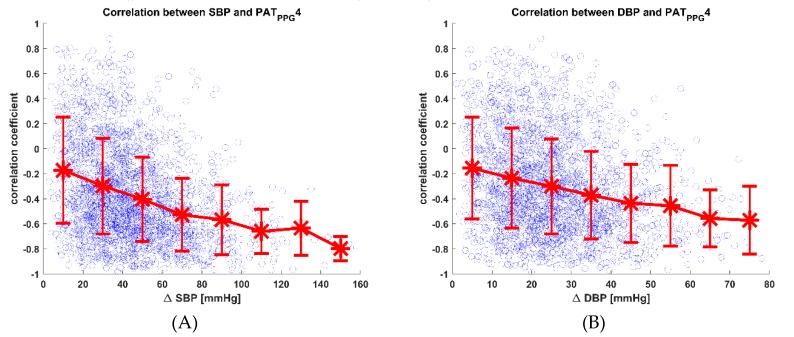
Scatter plot of correlation coefficients between BP and PAT_PPG_4 with respect to range of change of BP. ‘*’ symbols indicate the mean correlation coefficient for subjects in 20mmHg bins of ΔSBP or 10mmHg bins of ΔDBP, and vertical bars indicate the standard deviation of correlation coefficient values. (**A**) Scatter plot for correlation coefficients between SBP and PAT_PPG_4 with respect to ΔSBP; (**B**) Scatter plot for correlation coefficients between DBP and PAT_PPG_4 with respect to ΔDBP. SBP, systolic blood pressure; DBP, diastolic blood pressure; PAT, pulse arrival time; PPG, photoplethysmogram.

Figure 8 shows a scatter plot of the correlation between BP and PAT on the vertical axis and the range of change of BP (ΔBP) on the horizontal axis for all subjects. In general, the correlation between BP and PAT becomes more negative with increasing ΔBP, and the variance in the correlation values becomes smaller. This indicates that PAT acts as a better predictor of BP when there are large BP fluctuations, but may not be so ideal at lower BP fluctuations. With regards to NCBPM, the implications of this result may not be significant, as the accurate detection of small changes in BP are not critical as compared to the detection of larger changes in BP.

In principle, the correlation between BP and PAT should stay the same no matter the degree of hemodynamic change, but in practice this does not seem to hold true. There may be many reasons for this observation, but under the assumption that the correlation should be negative in most cases, large numbers of positive correlations at smaller ΔBP indicate that the changes in BP cannot be accounted for by the changes in PAT alone. Other possible reasons for the larger variation at lower ΔBP or lower ΔPAT may be attributed to technical factors (e.g., limitations in analog-to-digital conversion, as smaller changes in ΔPAT are harder to detect precisely at 100 Hz sampling rate), and other confounding factors (e.g., changes in cardiac output, which may have relatively larger influence on SBP when ΔPAT is small). However, considering that these factors were not measured in the current study, no definitive conclusions can be made with the available information, and a further study is warranted.

### 4.3. PAT as a Predictor of BP in Ubiquitous NCBPM

Although the DBP model developed here satisfies AAMI, BHS, and IEEE standards, upon close inspection, the model output does not follow the reference values in many cases. This can be seen in the correlation histograms (Figure 7), where the correlations between the estimated value and the reference value for some subjects are low or are negative. This indicates that although the model as a whole follows trends in BP change well enough and can satisfy the error criteria, its clinical use is not ideal, as the estimates for some subjects may provide misleading information endangering a patient in critical cases. Circadian fluctuations of BP leads to standard deviation between 12–25 mmHg for SBP and between 8–14 mmHg for DBP [12,58,59,60], which indicates that PAT_PPG_4 may not be an accurate predictor of BP for ubiquitous NCBPM purposes, as the range of change of BP is too small to guarantee that PAT has a high enough correlation to BP. Hence, a method beyond linear regression models is required, and a further study with more complex methods is warranted to develop a generalizable model of BP based on PAT.

### 4.4. Shortcomings of Current BP Monitor Standards with Regards to Beat-by-Beat NCBPM

For the data with large BP variations, high correlation between PAT and BP results in accurate BP estimates, but for the data with small BP variations, the range of BP are small enough such that even non-optimal predictors can produce satisfactory results with regards to the error in current BP monitor standards. This trend is seen in the error rates for DBP and SBP estimations where, although PAT has a more favorable correlation to SBP, the lower absolute magnitude of DBP led to smaller errors. This result brings into question the reliability of these BP device standards in relation to beat-by-beat blood pressure estimation. First, the error-based validation criteria do not reflect the individual cases where a given subject’s BP estimates are erroneous as long as the overall estimation performance in the sample population is acceptable. Second, these standards use a few points per subject and thus do not include a criterion for measuring similarity in trends between the reference and the estimate. For scenarios with the same absolute error and with opposite correlation values to the reference, these standards cannot distinguish the better case. For the subject with a negative correlation value but with acceptable limits of error, the results may be tolerable in the testing BP range, but if the same trend extrapolates to a larger BP range, the consequences could be dire. With these issues in mind, current standards should be updated to better determine the performance of the beat-by-beat NCBPM devices being developed. Without such updates, seemingly adequate devices which are unacceptable for clinical use may obtain the authentication marks bearing these standards.

### 4.5. Large Datasets for BP Estimation Model Development

NCBPM has gained wide attraction over the last decade and a growing number of methods are being developed for non-invasive BP estimation, but a probe into the published research reveals a key issue that researchers face in this area. As reported by Mukkamala et al. in a well-recognized review [27], most researchers tend to recruit a small number (*n* < 100) of homogeneous subjects (usually young healthy males) for the development of BP estimation algorithm based on features extracted from non-invasive biosignals. Furthermore, these studies also tend to induce BP change through a certain type of hemodynamic intervention and use oscillometry or the volume clamp method for reference BP measurement. These limitations extend to recent articles [61,62,63,64,65,66] beyond the ones covered in the review, and the validity of the methods developed with such limitations must be carefully assessed.

In the BHS, AAMI, and IEEE standards for BP monitors, performance in terms of errors are not the only set of criteria for validating BP monitors, and there are strict guidelines on subject selection as well as on the reference BP measurement. For example, validation study subjects must include both normotensive and hypertensive subjects with varying ranges of baseline BP, and the reference BP must be measured using intra-catheter BP or using auscultation with two separate observers who agree on the measured value. With reference to ubiquitous BP monitoring, it might be misguided to implement a BP estimation model developed using induced hemodynamic change, as BP change could arise from a wide range of causes. For example, a DBP estimation model developed from a normal healthy subject with induced BP variability from isotonic exercise will not be able to predict DBP changes of the same subject during isometric exercise due to the difference in the underlying physiological mechanisms that cause DBP change in isotonic and isometric exercises [67,68].

These limitations become even more restrictive if they must be considered collectively, and therefore it is unlikely that a practical NCBPM solution could be developed from such small-scale experiments. In light of this issue, there have been recent attempts to use large biosignal datasets to make BP models. Kachuee et al. used approximately 1000 unique subjects from the MIMIC dataset and achieved acceptable results for DBP [37], Wang et al. achieved more impressive results from the same database, but only used 72 subjects in total [39], and Ruiz-Rodriguez et al. developed their own PPG database of 572 subjects to estimate BP using deep neural networks, but the results were not clinically adequate [36]. In order to overcome the limitations mentioned above, the database used for BP estimation must be accessible and consistently reliable in terms of inter-waveform alignment, but both databases mentioned are lacking in these characteristics.

The database used in this study, the VitalDB, is the first openly available database that could be used to properly develop and validate BP estimation models using ECG and PPG. The VitalDB has biosignal data from patients undergoing various types of surgeries, and the data from these subjects can be used for training and validating BP estimation models. Due to its compliance to all the criteria mentioned above (i.e., a large heterogeneous subject pool with widely varying BP, reliable reference, and consistent biosignal measurement), it will be challenging to develop a generalizable BP model that satisfy the three BP monitor standards for both SBP and DBP using this dataset. Although a satisfactory DBP linear model was generated in this study, it is the authors’ hopes that the introduction of this dataset will lead to future studies with more complex approach to BP estimation, perhaps using deep neural networks, resulting in impressive DBP and SBP models that satisfy the standards and even overcome the limitations of these standards mentioned above.

### 4.6. Limitations

There are a few limitations to mention. First, there is a consistent delay between the signals due to the experimental setup. The exact data collection logic is not known, but the average PAT_PPG_4 was around 650 ms, which is extremely large compared to previous reports of PAT from ECG to finger PPG. Although PAT_PPG_ varies largely based on the point on PPG at which PAT is extracted, this seems to be quite large compared to PAT_ABP_ which averages around 150 ms. Comparing these values to previous studies with similar setups, it is likely that there exists a delay between ECG, ABP, and PPG. However, this delay was consistent in the entire dataset with intrasubject PAT_PPG_4 mean ranging between 600 ms to 670 ms, and intersubject mean at 645 ms and standard deviation at 26 ms, which shows a general shift of the PAT value from the expected range around 300 ms. Due to the presence of this offset, the authors have verified with the creators of the database the consistency of this delay throughout all subject recordings. Hence, due to the uniform protocol in the measurement of the signals in this dataset, this delay is consistent and does not affect the analyses we have performed in this study.

The second limitation of this study was the variation in the surgery types used in this study. Although the wide range of surgeries may represent wide sources of hemodynamic change, it also represents a source of inconsistency in analyses. Different types of surgeries with different anesthesia call for an in-depth analysis based on anesthesia use and subsequent BP change, but this could not be done as the timing for the dosing was not exact according to the authors of the database. Furthermore, certain recordings may include hemorrhage and subsequent blood transfusion events which would distort pulse wave transmission, making the data unacceptable for some of the analyses above. However, by pre-processing the dataset with visual inspection, we believe we have eliminated many of the recordings or parts of recordings with low SNR, and we have performed the analyses above without taking the type of surgery into consideration as any dataset with such a large subject pool would have sources of variations that cannot be accounted for. Additionally, with regards to the surgical setup of each subject recording, varying the arm positions could have an important confounding effect on the measurement of PAT or PTT and hence the analyses in the present study. However, the information on the arm positions were not recorded, and this confounder could not be taken into account during the analyses.

Lastly, the number of data per subject averaged around 3000 points, but the actual number of data per subject was different, ranging from 101 points to 22,876 points. However, the same analyses with 100 points per subject yielded similar results with a non-significant difference in the results. In future studies which use this dataset to develop BP estimation models, the number of data points per subject should be matched for proper validation.

## Figures and Tables

**Table 1:** Demographic and BP characteristics of the data (*N* = 2309).

Characteristics	Subjects (*N* = 2309)
Age (yrs)	58 ± 15 (range 5–92)
Gender (male)	1218 (53%)
Height (cm)	162 ± 9
Weight (kg)	61 ± 12
BMI (kg/m^2^)	23 ± 4
Hypertension	777 (34%)
Diabetes	262 (11%)
Arrhythmia	13 (1%)
# of features	2935 ± 2226
SBP	
Mean value (mmHg)	116 ± 15
Δ· value * (mmHg)	44 ± 23
DBP	
Mean value (mmHg)	63 ± 10
Δ· value * (mmHg)	26 ± 13
MBP	
Mean value (mmHg)	82 ± 11
Δ· value * (mmHg)	33 ± 17
PP	
Mean value (mmHg)	54 ± 12
Δ· value * (mmHg)	25 ± 14

BMI, body mass index; #, number; SBP, systolic blood pressure; DBP, diastolic blood pressure; MBP, mean blood pressure; PP, pulse pressure. * The difference between max and min values of each recordings.

**Table 2:** Correlation analysis result of the data (*N* = 2309).

	PAT_ABP_	PAT_PPG_1	PAT_PPG_2	PAT_PPG3_	PAT_PPG_4	PTT1	PTT2	PTT3	PTT4
SBP	−0.46 ± 0.35	−0.22 ± 0.40	−0.24 ± 0.43	−0.35 ± 0.36	−0.37 ± 0.37	−0.04 ± 0.39	−0.01 ± 0.41	−0.11 ± 0.36	−0.12±0.37
DBP	−0.35 ± 0.38	−0.18 ± 0.40	−0.19 ± 0.42	−0.28 ± 0.37	−0.30 ± 0.38	−0.05 ± 0.38	−0.01 ± 0.40	−0.10 ± 0.36	−0.11±0.37
MBP	−0.42 ± 0.36	−0.21 ± 0.40	−0.21 ± 0.43	−0.32 ± 0.37	−0.34 ± 0.38	−0.05 ± 0.39	−0.01 ± 0.40	−0.11 ± 0.36	−0.12±0.37
PP	−0.50 ± 0.34	−0.23 ± 0.39	−0.25 ± 0.42	−0.36 ± 0.35	−0.39 ± 0.37	−0.04 ± 0.38	−0.01 ± 0.40	−0.11 ± 0.35	−0.11±0.36

PAT, pulse arrival time; PTT, pulse transit time; ABP, arterial blood pressure; SBP, systolic blood pressure; DBP, diastolic blood pressure; MBP, mean blood pressure; PP, pulse pressure.

**Table 3:** Performance of population BP linear model (*N* = 2309).

Performance Measure	SBP	DBP
Correlation to reference	0.37 ± 0.37	0.30 ± 0.38
ME (mmHg)	<0.001	<0.001
SDE (mmHg)	11.04	6.25
Cumulative Error < 5 mmHg (%)	41.6	65.7
Cumulative Error < 10 mmHg (%)	69.7	89.8
Cumulative Error < 15 mmHg (%)	85.0	97.0
MAD (mmHg)	8.21	4.58

BP, blood pressure; SBP, systolic blood pressure; DBP, diastolic blood pressure; ME, mean error; SDE, standard deviation of the error; MAD, mean absolute difference.

**Table 4:** Multivariate regression analysis of risk factors (*N* = 2309).

	Slope between PAT_PPG_4 and SBP				Correlation between PAT_PPG_4 and SBP			
	β	SE	*P*-Value	VIF	β	SE	*P*-Value	VIF
Age	−0.004	0.001	0.000 *	1.145	−0.001	0.001	0.165	1.145
Gender	−0.016	0.020	0.411	1.015	−0.014	0.016	0.370	1.015
BMI	0.001	0.003	0.812	1.032	0.001	0.002	0.722	1.032
Hypertension	−0.017	0.022	0.442	1.157	−0.003	−0.004	0.860	1.157
Diabetes	−0.034	0.031	0.281	1.041	−0.03	−0.025	0.233	1.041

* *P* < 0.05 for *t*-test of the regression coefficient. β, parameter estimate (β coefficient); SE, standard error; VIF, variance influence factor; PAT, pulse arrival time; SBP, systolic blood pressure; BMI, body mass index.

## Supplementary Materials

The following are available online at https://www.mdpi.com/2077-0383/8/11/1773/s1, Figure S1: Bland-Altman plots of the errors between the estimated and reference BP of 2309 subjects, Table S1: Parameter characteristics of the data (*N* = 2309).
