# Supplementary material for: Analysis of Pulse Arrival Time as an Indicator of Blood Pressure in a Large Surgical Biosignal Database: Recommendations for Developing Ubiquitous Blood Pressure Monitoring Methods
Source: J Clin Med. 2019 Oct 24;8(11):1773. doi: 10.3390/jcm8111773 (PMC6912522; doi:10.3390/jcm8111773)
Supplement: Supplementary file 1 [file jcm-08-01773-s001.pdf]

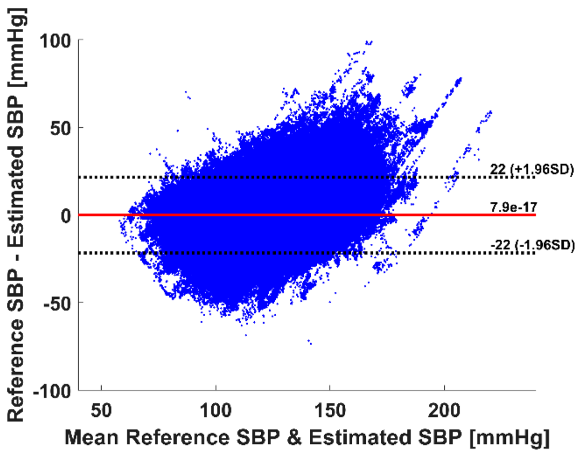

(**A**)

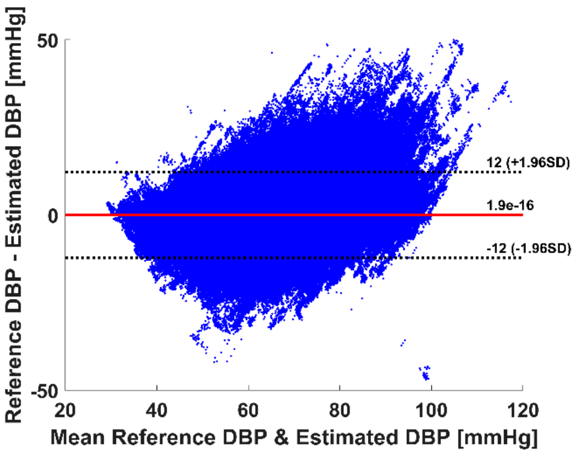

(**B**)

Figure S1. Bland-Altman plots of the errors between the estimated and reference BP of 2309 subjects. (A) Bland-Altman plots for SBP estimation; (B) Bland-Altman plots for DBP estimation. SBP, systolic blood pressure; DBP, diastolic blood pressure.

Table S1. Parameter characteristics of the data (*N* = 2309).

	**PAT_{ABP}**	**$PAT_{PPG}1$**	**$PAT_{PPG}2$**	**$PAT_{PPG}3$**	**$PAT_{PPG}4$**	**PTT1**	**PTT2**	**PTT3**	**PTT4**
**Mean value [ms]**	157±21	590±36	802±32	717±26	645±26	433±31	644±29	559±21	499±20
**Δ value* [ms]**	40±20	97±55	68±32	62±24	55±25	97±53	71±31	63±23	57±23

PAT, pulse arrival time; PTT, pulse transit time; ABP, arterial blood pressure; PPG, photoplethysmogram; * The difference between max and min values of each recordings.
